# Study of *lug* Operon, SCC*mec* Elements, Antimicrobial Resistance, MGEs, and STs of *Staphylococcus lugdunensis* Clinical Isolates Through Whole-Genome Sequencing

**DOI:** 10.3390/ijms26136106

**Published:** 2025-06-25

**Authors:** Tein-Yao Chang, Lee-Chung Lin, Cheng-Yen Kao, Jang-Jih Lu

**Affiliations:** 1 Institute of Preventive Medicine, National Defense Medical Center, New Taipei City 114, Taiwan; ttyaoc@ndmctsgh.edu.tw; 2 Graduate Institute of Pathology and Parasitology, National Defense Medical Center, Taipei 114, Taiwan; 3 Department of Laboratory Medicine, Linkou Chang Gung Memorial Hospital, Taoyuan 333, Taiwan; leollc@gmail.com; 4 Institute of Microbiology and Immunology, National Yang Ming Chiao Tung University, Taipei 112, Taiwan; kaocy@nycu.edu.tw; 5 Division of Clinical Pathology, Taipei Tzu Chi Hospital, Buddhist Tzu Chi Medical Foundation, New Taipei City 231, Taiwan

**Keywords:** *Staphylococcus lugdunensis*, lugdunin, MLST, SCC*mec*, IS256

## Abstract

*Staphylococcus lugdunensis* is a coagulase-negative staphylococcus known for its significant pathogenic potential, often causing severe infections such as endocarditis and bacteremia, with virulence comparable to *S. aureus*. Despite general susceptibility to most antibiotics, the emergence of oxacillin-resistant strains is increasingly concerning. This study conducted whole-genome sequencing on 20 *S. lugdunensis* isolates from Chang Gung Memorial Hospital to explore their genetic diversity, antimicrobial resistance mechanisms, and mobile genetic elements. The lugdunin biosynthetic operon, essential for antimicrobial peptide production, was present in multilocus sequence typing (MLST) types 1, 3, and 6 but absent in STs 4, 27, and 29. Additionally, IS256 insertion elements, ranging from 7 to 17 copies, were identified in four strains and linked to multidrug resistance. CRISPR-Cas systems varied across STs, with type III-A predominant in ST1 and ST6 and type IIC in ST4, ST27, and ST29; notably, ST3 lacked CRISPR systems, correlating with a higher diversity of SCC*mec* elements and an increased potential for horizontal gene transfer. Phage analysis revealed stable phage–host associations in ST1, ST6, and ST29, whereas ST4 displayed a varied prophage profile. Phenotypic resistance profiles generally aligned with genomic predictions, although discrepancies were observed for aminoglycosides and clindamycin. These findings highlight the complex genetic landscape and evolutionary dynamics of *S. lugdunensis*, emphasizing the need for genomic surveillance to inform clinical management and prevent the spread of resistant strains.

## 1. Introduction

*S. lugdunensis* is a coagulase-negative staphylococcus (CoNS) known for its clinical significance, particularly in nosocomial infections [1]. While traditionally considered part of the human skin microbiota, *S. lugdunensis* has emerged as an opportunistic pathogen, causing severe infections such as endocarditis, bacteremia, and skin and soft tissue infections [2]. Unlike other CoNS, *S. lugdunensis* exhibits virulence comparable to *S. aureus* and causes infective endocarditis in a similar proportion of cases as *S. aureus* [3]. Despite its virulence potential, *S. lugdunensis* remains largely susceptible to most antibiotics, though oxacillin-resistant *S. lugdunensis* (ORSL) strains are becoming more prevalent in hospital settings [4].

Multilocus sequence typing (MLST) and pulsed-field gel electrophoresis (PFGE) have been valuable genotypic tools for analyzing the genetic relatedness and population structure of *S. lugdunensis* isolates. MLST, in particular, has been widely used to classify isolates into sequence types (STs) by analyzing the allelic profiles of seven housekeeping genes [5]. PCR-based assays targeting the *mecA* gene are essential for detecting oxacillin-resistant strains of *S. lugdunensis* (ORSL), as the presence of the *mecA* gene generally confers resistance to beta-lactam antibiotics [6,7]. Furthermore, staphylococcal cassette chromosome *mec* (SCC*mec*) typing through PCR is crucial for determining the SCCmec elements that harbor the *mecA* gene. Although these traditional methods have proven reliable, they are increasingly being complemented by whole-genome sequencing (WGS) for more comprehensive analysis of genetic variation and resistance determinants in *S. lugdunensis* [8].

Whole-genome sequencing (WGS) has revolutionized our understanding of the genetic landscape of bacterial pathogens, providing insights into virulence factors, antimicrobial resistance, and horizontal gene transfer mechanisms [9,10]. For *S. lugdunensis*, WGS studies have revealed a closed pan-genome with significant barriers to horizontal gene transfer, including restriction–modification systems and CRISPR-Cas loci. Mobile genetic elements (MGEs), such as SCC*mec*, plasmids, and prophages, play a crucial role in the dissemination of antimicrobial resistance and virulence genes [11,12,13]. In particular, SCC*mec* elements are responsible for conferring methicillin resistance in *S. lugdunensis*, with SCC*mec* types II and V or Vt being prevalent in clinical isolates [4,7,12].

The *lug* operon is a biosynthetic gene cluster responsible for producing lugdunin, a cyclic peptide antibiotic with potent antimicrobial activity. First identified in *S. lugdunensis*, the *lug* operon comprises four non-ribosomal peptide synthetase (NRPS) genes, *lugA*, *lugB*, *lugC*, and *lugD*, which encode the machinery required for lugdunin biosynthesis [2,14]. The *lug* operon plays a critical role in the biosynthesis of lugdunin, a bacteriocin-like antimicrobial peptide that has been shown to inhibit *S. aureus* colonization in the nasal microbiome [15]. Given its antimicrobial properties, the presence and regulation of the *lug* operon may influence the evolutionary dynamics of *S. lugdunensis*, particularly in the context of horizontal gene transfer (HGT) events involving mobile genetic elements (MGEs). Previous studies have indicated that MGEs, including SCC*mec* and IS256, facilitate the dissemination of antibiotic resistance genes and virulence factors among *Staphylococcus* species [12]. Therefore, analyzing the *lug* operon alongside these MGEs provides insights into whether lugdunin production influences the acquisition or retention of antibiotic resistance determinants. Additionally, the regulatory interplay between *lug* operon expression and antimicrobial resistance could provide a better understanding of how *S. lugdunensis* adapts to different ecological niches.

The insertion sequence element IS256 is a well-studied transposable element known to contribute significantly to genome plasticity, the regulation of antimicrobial resistance, and adaptation in *Staphylococcus* species. In *S. aureus*, IS256 is often associated with SCCmec-mediated β-lactam resistance and has been implicated in the inactivation of regulatory genes involved in biofilm formation and virulence [16,17]. Horizontal gene transfer (HGT) has facilitated the spread of IS256 from *S. aureus* to coagulase-negative staphylococci (CoNS), including *S. epidermidis* and *S. lugdunensis*. The movement of IS256 between species suggests that it may contribute to genetic adaptation in *S. lugdunensis*, particularly in hospital-associated clones that exhibit multidrug resistance. To better understand this process, we investigated the distribution of IS256 in *S. lugdunensis* isolates and examined its potential role in SCCmec mobility, antibiotic resistance modulation, and other genomic changes that could impact bacterial fitness.

In this study, we focused on the *lug* operon, SCC*mec* elements, STs, and the distribution of MGEs, comparing our findings to traditional PCR-based methods and MLST. Understanding the genomic architecture of these isolates, including the presence of MGEs and their role in antimicrobial resistance, will provide valuable insights into the evolutionary dynamics of *S. lugdunensis* and its clinical implications in healthcare settings.

## 2. Results

### 2.1. Lugdunin Biosynthetic Cassette in 20 S. lugdunensis Strains

**Figure 1:**
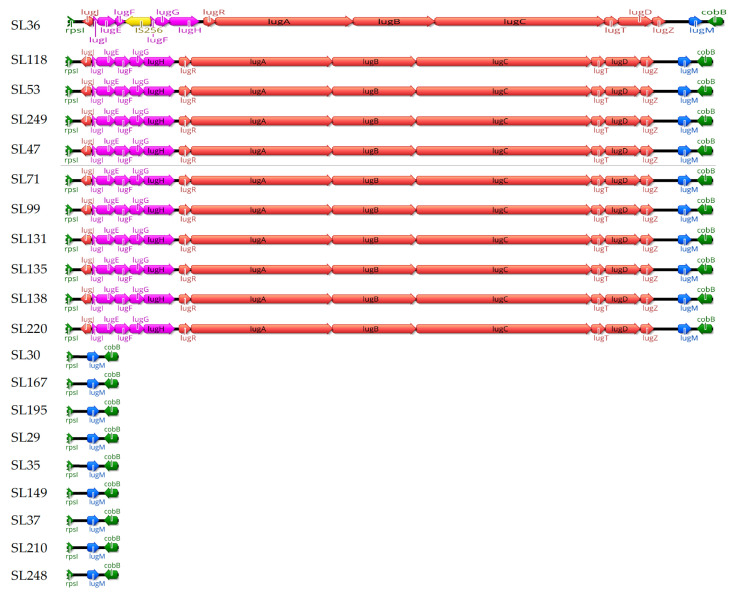
Structural organization of the lugdunin cassette between the *rpsL* and *cobB* genes. This figure shows the structural variation in the lugdunin (Lug) cassette located between the *rpsI* (**left**) and *cobB* (**right**) genes (green) across 20 strains. Eleven strains (SL36, SL118, SL249, SL131, SL138, SL220, SL99, SL135, SL47, SL71, and SL53) possess the full Lug cassette, while nine strains (SL195, SL29, SL35, SL37, SL167, SL248, SL30, SL149, and SL210) contain only the *lugM* gene (blue). An IS256 insertion (yellow) is observed in strain SL36, which interrupts the *lugF* gene. Genes encoding non-ribosomal peptide synthetase (NRPS) components (*lugA–E*) are colored red, and genes encoding the ABC transporter system (*lugF–H*) are colored pink.

The distribution of the lugdunin biosynthetic cassette varied across the 20 *S. lugdunensis* strains. The complete operon, comprising the genes *lugA*, *lugB*, *lugC*, and *lugD*, was identified in strains belonging to ST1 (SL53, SL249), ST3 (SL47, SL71, SL99, SL131, SL135, SL138, SL220), and ST6 (SL36, SL118). In contrast, strains from ST4 (SL30, SL167, and SL195), ST27 (SL29, SL35, SL149, SL37, and SL210), and ST29 (SL248) lacked the operon and the associated *lugE*, *lugF*, *lugG*, and *lugH* genes. These genes encode the non-ribosomal peptide synthetase (NRPS) complex and components of an ABC transporter essential for lugdunin synthesis (Figure 1).

Notably, an IS256 insertion was identified in strain SL36, disrupting the *lugF* gene, which encodes a membrane-associated component of a putative ABC transporter involved in lugdunin export [18].

### 2.2. IS256 Insertion Sequence in S. lugdunensis Genome

**Figure 2:**
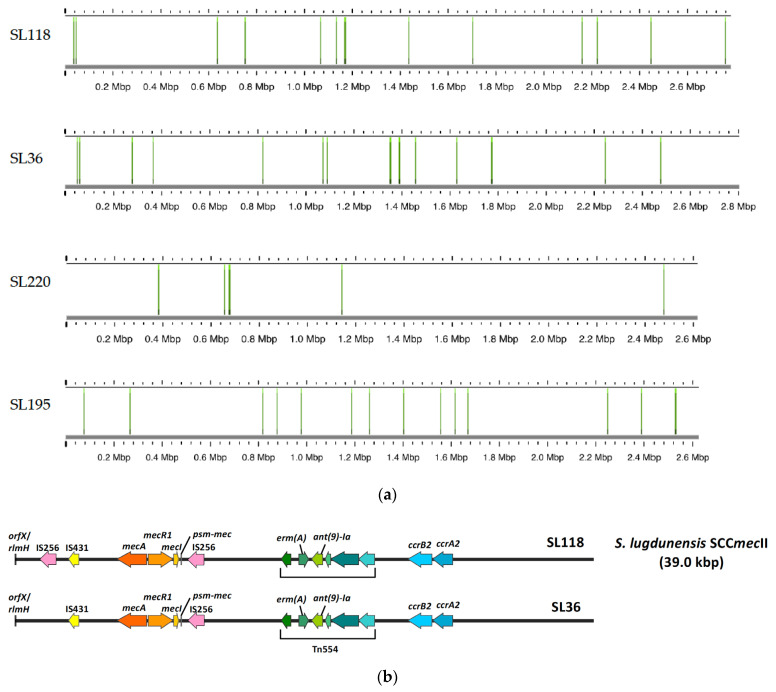
IS256 copy numbers and specific insertion locations in four strains genome. (**a**) This figure illustrates the copy numbers of IS256 elements and the specific locations of these insertions in four strains: SL36, SL118, SL195, and SL220. Strain SL36 contains 17 copies of IS256. SL118 has 14 copies. SL195 contains 15 copies, and SL220 harbors 7 copies. For the remaining strains, no IS256 elements were detected. (**b**) This figure shows the structural organization of the SCC*mec* type II cassette (39.0 kbp) in *S. lugdunensis* strains SL118 and SL36. In strain SL118, two IS256 elements are present: one located near the *mecI* gene and the other adjacent to the IS431 element. In contrast, strain SL36 contains a single IS256 insertion located close to the *mecI* gene.

The analysis of IS256 elements across 20 *S. lugdunensis* strains revealed that IS256 was present in only four strains: SL36, SL118, SL220, and SL195 (Figure 2). Strains SL36, SL118, SL220, and SL195 contain 17 copies, 7 copies, 14 copies, and 15 copies of IS256, respectively. No IS256 elements were detected in the remaining 16 strains. An examination of the SCC*mec* type II cassette structure in SL118 and SL36 shows that SL118 has two IS256 insertions—one near the *mecI* gene and another adjacent to the IS431 element—whereas SL36 contains a single IS256 insertion located near the *mecI* gene. This variability in IS256 copy numbers and insertion locations suggests a diverse impact on the genomic architecture of these strains.

### 2.3. CRISPR-Cas Systems in S. lugdunensis Strains

CRISPR-Cas systems were detected in 8 out of 20 *S. lugdunensis* strains, and their presence showed a clear association with specific sequence types (STs) (Table 1). Type III-A CRISPR-Cas systems were identified in ST1 (SL53 and SL249) and ST6 (SL36 and SL118), while type IIC systems were found in ST4 (SL30, SL167, and SL195), ST27 (SL29, SL35, SL149, SL37, and SL210), and ST29 (SL248).

In contrast, all ST3 strains (SL47, SL71, SL99, SL131, SL135, SL138, and SL220) lacked detectable CRISPR-Cas systems. The ST3 group also exhibited the highest diversity in SCC*mec* elements; however this diversity did not coincide with an increase in mobile elements such as IS256, which was detected in one ST3 strain.

### 2.4. Phage Sequence Distribution by Sequence Type

Prophage analysis revealed notable variation among *S. lugdunensis* strains (Table 2). Intact phage sequences were detected in strains from ST1 (SL53), ST4 (SL30, SL167, and SL195), ST6 (SL36 and SL118), and ST29 (SL248). In contrast, most ST3 strains lacked detectable prophages, except for SL71, which carried a 47.9 kb intact phage like *Staphylococcus* phage StB20.

ST6 strains exhibited the most complex phage profiles, each harboring two phages including large SPbeta-like elements. ST4 strains also showed multiple phage sequences with varying completeness. ST27 strains carried only incomplete prophages or none.

The most frequently matched phage was *Staphylococcus* PT1028, followed by SPbeta-like and CNPx-related phages. The GC content ranged from 29.6% to 35.4%, consistent with that of the host genome. Prophage profiles varied among sequence types and were highly strain specific.

### 2.5. MLST Types and SCCmec Elements in S. lugdunensis

The data highlights the relationship between MLST types and SCC*mec* elements in various *S. lugdunensis* strains (Table 1). Strains belonging to ST1 (SL53 and SL249) and ST4 (SL30, SL167, and SL195) do not carry any SCC*mec* elements and are susceptible to oxacillin. Type ST3 strains, including SL47, SL71, SL99, and SL131, are associated with SCCmec type V, while SL135 carries a variant form, SCCmec type Vt. However, the SCCmec types of these strains do not consistently correlate with their oxacillin susceptibility profiles—SL47, SL99, and SL131 exhibit resistance to oxacillin, whereas SL71 and SL135 remain susceptible. Strain SL138, also an ST3 strain, harbors SCC*mec* type IV and exhibits oxacillin resistance, while SL220, another ST3 strain, does not carry SCC*mec* elements and remains oxacillin-susceptible. Type ST6 strains (SL36 and SL118) carry SCC*mec* type II but remain oxacillin-susceptible.

ST27 strains, such as SL29 and SL35, are associated with SCC*mec* type V but remain susceptible to oxacillin, while SL149 carries SCC*mec* type Vt and is also resistant. In contrast, the type ST27 strains SL37 and SL210 lack SCC*mec* elements and are oxacillin-susceptible. Lastly, strain SL248, belonging to type ST29, does not carry any SCC*mec* elements and is susceptible to oxacillin.

### 2.6. Antibiotic Resistance in S. lugdunensis

The antimicrobial resistance (AMR) profiles of *S. lugdunensis* strains, based on disk diffusion testing, are largely consistent with the WGS predictions from ResFinder. However, some discrepancies merit discussion. For β-lactam antibiotics, strains such as SL47, SL99, SL131, SL135, SL138, SL36, and SL118 exhibit resistance to penicillin and oxacillin, which correlates well with the presence of the *mecA* and *blaZ* genes identified by WGS (Table 3). In contrast, strains SL53 and SL249, which lack these resistance genes, show susceptibility to both antibiotics, as predicted by WGS. Strain SL71 harbored both mecA and blaZ but was susceptible to oxacillin, with a minimum inhibitory concentration (MIC) of 0.5 μg/mL determined by broth dilution (Supplementary Table S1).

Among the five strains resistant to both erythromycin and clindamycin, three (SL36, SL118, and SL248) carried the erm(A) gene in combination with other resistance determinants (Table 3). Two additional strains (SL138 and SL220) were resistant to both antibiotics but lacked *erm*(*A*), instead harboring alternative resistance genes such as *blaZ* and *dfrG*. The remaining 13 strains were phenotypically susceptible to both erythromycin and clindamycin, with no *erm*(*A*) detected in these isolates. Most of these strains (*n* = 10) also lacked any resistance genes, except for low-prevalence genes such as *blaZ* and *mecA*. Interestingly, SL210 exhibited resistance to erythromycin while remaining phenotypically sensitive to clindamycin and lacked detectable resistance genes.

Similarly, for trimethoprim–sulfamethoxazole (SXT), most strains are susceptible, except for SL220, which shows resistance both phenotypically and genotypically due to the presence of the *dfrG* gene.

A notable discrepancy is observed with aminoglycosides. Strains SL36 and SL118 exhibit phenotypic resistance to gentamicin, tobramycin, and streptomycin, which correlates with the presence of aminoglycoside-modifying genes (*aac*(*6′*)-*aph*(*2″*), *aph*(*3′*)-*III*, and *ant*(*9*)-*Ia*) (Table 3). However, SL195, despite possessing the *aac*(*6′*)-*aph*(*2″*) gene, shows susceptibility to aminoglycosides, suggesting that the resistance gene might not be expressed or other factors might influence phenotypic resistance. Furthermore, all strains are phenotypically susceptible to tetracycline and vancomycin, which aligns with the absence of resistance genes in the WGS data.

## 3. Discussion

This study investigated the presence and structural variation in the lugdunin biosynthetic cassette, IS256 insertion elements, and AMR profiles across 20 *S. lugdunensis* strains, alongside an analysis of their CRISPR-Cas systems, SCC*mec* elements, and phage interactions. Our findings offer novel insights into the genetic diversity of *S. lugdunensis*, revealing lineage-specific variations and evolutionary mechanisms that may influence both pathogenicity and antibiotic resistance.

The identification of the lugdunin biosynthetic cassette (*lugA*-*lugD*) in strains from STs 1, 3, and 6 aligns with previous studies showing that lugdunin production is often restricted to specific lineages [15]. This production may confer a competitive advantage in microbial communities such as the human nasal cavity [19]. Lugdunin has potent antimicrobial activity against *S. aureus* and other pathogens, supporting the hypothesis that its production by *S. lugdunensis* plays a crucial role in the strain’s survival in polymicrobial environments [2]. The *lug* operon, essential for lugdunin biosynthesis, includes genes involved in the non-ribosomal peptide synthetase (NRPS) pathway [15]. However, in strains from STs 4, 27, and 29, the full *lug* operon is absent, likely due to evolutionary divergence. Despite the loss of the full operon, these strains retain *lugM*, which encodes a putative monooxygenase. This observation is consistent with prior reports from China showing that certain strains (RMLUG2, VCU150, VISLISI_25, and C_33) only carried *lugM* [20]. Although the role of *lugM* in the biosynthesis process remains unclear, it may serve a regulatory or vestigial function [21].

The analysis of IS256 insertion elements revealed copy numbers ranging from 7 to 17 among selected strains. IS256 was present not only in ST3 (which lack CRISPR-Cas systems) but also in ST4 and ST6 (which possessed CRISPR-Cas IIC and CRISPR-Cas III-A, respectively), suggesting that CRISPR-Cas systems may not restrict IS256 proliferation. Further research with additional strains is necessary to confirm this observation. Prior studies, such as those by Kozitskaya et al., have shown that multiple copies of IS256 are common in the genomes of multi-resistant *S. epidermidis* and are associated with resistance to antibiotics like gentamicin and oxacillin [16]. Similarly, IS256 and IS257R2 have been found in osteomyelitis-causing MRSA isolates [22], and its association with biofilm formation and multidrug resistance has been documented in *S. aureus* [17]. Our results indicate a similar trend in strains SL36, SL118, and SL220, which harbored high IS256 copy numbers and are multidrug-resistant. However, strain SL195, despite also carrying multiple IS256 copies, remained phenotypically susceptible, implying that additional regulatory mechanisms may influence resistance expression. Notably, in SL36, an IS256 insertion was located within the *lugF* gene, which is part of the *lugEFGH* cassette. Functional studies have shown that the deletion of *lugEF* significantly reduces lugdunin export, highlighting its central role in transporter function [18]. Therefore, the disruption of *lugF* by IS256 in SL36 may impair lugdunin secretion, potentially affecting its antimicrobial capacity and competitive fitness.

CRISPR-Cas systems, which are known for their roles in defense against bacteriophages and limiting horizontal gene transfer, were analyzed in the *S. lugdunensis* strains. In clinical MRSA strains from Denmark, only 2.9% were found to carry CRISPR-Cas systems, with type III-A CRISPR-Cas, located within the SCCmec type V (5C2&5) cassette, being the most common [23]. In *S. lugdunensis*, type III-A CRISPR-Cas was found to be dominant, followed by type IIC CRISPR-Cas systems [24].

Type III-A CRISPR-Cas systems were identified in ST1 and ST6 strains, while type IIC systems were found in ST1, ST4, ST27, and ST29. In contrast, ST3 strains lacked CRISPR-Cas systems and displayed greater SCCmec diversity, suggesting that the absence of CRISPR immunity may facilitate horizontal gene transfer and the acquisition of mobile resistance elements [25].

According to Chassain et al., both ST1 and ST6 are members of clonal complex 1 (CC1), with ST6 representing the ancestral genotype [26]. In our dataset, ST6 strains were penicillin-resistant, whereas ST1 strains were penicillin-susceptible, consistent with previous reports for ST1 and ST6. These observations support the notion that the clonal background influences both antimicrobial resistance profiles and the distribution of accessory genome elements. Moreover, the presence of type III-A CRISPR-Cas systems in both ST1 and ST6 may reflect vertical inheritance within CC1, with retention in specific descendant lineages.

Notably, we observed the absence of CRISPR-Cas systems in ST3 strains, which exhibited greater diversity in SCCmec elements, suggesting that CRISPR-Cas deficiency may facilitate horizontal gene transfer, potentially increasing the acquisition of antibiotic resistance genes. Similar findings were observed in *S. aureus*, where CRISPR-Cas deficiency is associated with higher rates of gene acquisition and a greater prevalence of mobile genetic elements [25].

In a genomic study of *S. lugdunensis*, approximately 33% of the strains were found to contain a functional CRISPR-Cas Type III-A system [24], which may contribute to limiting gene exchange [27]. However, CRISPR-Cas systems are not prevalent across all staphylococcal species. For instance, Abdullahi et al. (2024) found that only 19.2% of multidrug-resistant coagulase-negative staphylococci (CoNS) strains, including species such as *S. borealis* and *S. epidermidis*, harbored a CRISPR-Cas system [28]. This low prevalence supports the notion that the absence of CRISPR-Cas may promote horizontal gene transfer, potentially increasing the acquisition of antibiotic resistance genes in these species.

Prophage distribution, which remains underexplored in *S. lugdunensis*, also varied among the strains analyzed in this study. The presence of a CRISPR-Cas system is often inversely correlated with prophage abundance [29], as CRISPR-Cas targets and disables phage DNA before integration into the genome [30]. Most ST3 strains (SL47, SL99, SL131, SL135, and SL138) lacked detectable phages, suggesting either a loss of phages or failure to acquire them. However, SL71, another ST3 strain, carried a large, intact phage, indicating that phage acquisition is still possible despite the absence of CRISPR-Cas systems. This variability within the same sequence type highlights the complexity of phage–host interactions. ST27 strains exhibited a mixed pattern: some (SL29 and SL35) harbored incomplete phages, while others (SL37, SL149, and SL210) lacked phages entirely, suggesting partial retention or the complete loss of prophages. The absence of prophages in most ST3 strains, despite their lack of CRISPR-Cas systems, suggests that other evolutionary factors may influence phage acquisition, potentially driven by selective pressures for genomic stability or alternative defense mechanisms. These findings indicate that prophage carriage in *S. lugdunensis* is both sequence type-dependent and highly strain-specific, suggesting that phage–host interactions may contribute to genomic differentiation among lineages.

In this study, we also identified discrepancies between the presence of SCCmec elements and phenotypic oxacillin resistance. For example, strains SL29 and SL35 harbored SCCmec type V but remained oxacillin-susceptible. This observation aligns with findings by Ho et al. [31], who reported mecA-positive *S. lugdunensis* isolates carrying SCCmec type V that were phenotypically susceptible yet expressed mecA, as confirmed by a PBP2a immunoassay. Importantly, Ho et al. also highlighted that current disk (cefoxitin)- and MIC-based breakpoints may not reliably detect oxacillin resistance in such strains, especially within the ST27 lineage [31]. These findings emphasize that SCCmec carriage does not necessarily confer phenotypic resistance and that gene expression, cassette functionality, or regulatory mechanisms may critically influence resistance expression.

Our study also compared phenotypic antibiotic resistance profiles with genotypic predictions based on genome sequencing. For example, strains SL36 and SL118 exhibit resistance to multiple antibiotics, consistent with the detection of genes like *mecA* and aminoglycoside-modifying enzymes (*aac*(*6′*)-*aph*(*2″*)). However, some strains showed discrepancies between their phenotypic and genotypic resistance, which suggests that factors like gene regulation, expression levels, or compensatory mutations may impact resistance expression. The complex resistance patterns observed in some strains, involving SCC*mec* elements and β-lactamase genes, emphasize the intricate nature of antibiotic resistance.

In summary, WGS of *S. lugdunensis* is a powerful tool for evaluating antibiotic resistance, strain diversity, and the genetic mechanisms underlying multidrug resistance. WGS provides detailed insights into resistance genes and mobile genetic elements like SCCmec and prophages. Nevertheless, further investigation is required to explore uncharacterized regulatory mechanisms and other genetic factors to fully understand the bacterial resistance and pathogenic potential.

## 4. Materials and Methods

### 4.1. Bacterial Strains

A total of 20 *S. lugdunensis* isolates were collected between 2010 and 2014 from Chang Gung Memorial Hospital (Linkou), Taiwan. All *S. lugdunensis* isolates were identified using a Bruker Biotyper (database 2.0) matrix-assisted laser desorption ionization-time-of-flight mass spectrometry system (Bruker Daltonics, Bremen, Germany), according to the manufacturer’s instructions, and *S. lugdunensis* isolates were stored in a tryptic soy broth with 20% glycerol at −80 °C until further experiments. All confirmed strains were cultured on Brain-Heart Infusion (BHI) agar at 37 °C for 18–24 h. The antibiotic susceptibility of the strains were assessed using the disk diffusion method according to the recommendations of the Clinical and Laboratory Standards Institute [32], covering a range of antibiotics including penicillin (P), cefoxitin (FOX), clindamycin (CC), erythromycin (E), trimethoprim–sulfamethoxazole (SXT), and teicoplanin (TEC). Vancomycin (VA) susceptibility was evaluated using the agar dilution method. In this study, isolates with a vancomycin MIC of <6 μg/mL were considered susceptible based on internal laboratory criteria. We acknowledge that this interpretive threshold differs from the CLSI M100 breakpoint for non-*aureus Staphylococcus* spp., which defines susceptibility as ≤4 μg/mL and resistance as ≥32 μg/mL. This difference should be considered when comparing our results with studies that strictly follow CLSI breakpoints. A summary of the specimen sources from which the isolates originated (e.g., blood, pus, and wound) is provided in Supplementary Table S1.

### 4.2. Multilocus Sequence Typing and SCCmec Typing

To characterize the sequence types and SCCmec backgrounds of the isolates, MLST and SCCmec typing were performed prior to whole-genome sequencing on all 20 *S. lugdunensis* strains. Bacterial genomic DNA was extracted using the QIAamp DNA Mini Kit (Qiagen, Hilden, Germany), following the manufacturer’s protocol, with lysostaphin treatment to enhance cell wall lysis. SCCmec typing was performed by multiplex PCR based on previously described protocols [33], and MLST was conducted by sequencing seven housekeeping genes (*aroE*, *dat*, *ddl*, *gmk*, *ldh*, *recA*, and *yqiL*) [26]. Sequence types (STs) were determined using the MLST database (https://bigsdb.pasteur.fr/, accessed on 10 September 2024). MLST and SCCmec results were also cross-validated by Sanger sequencing to confirm the accuracy of Nanopore-based genome assemblies.

### 4.3. Bioinformatic Analysis

Whole-genome sequencing of 20 *Staphylococcus lugdunensis* isolates was performed using either the Oxford Nanopore MinION Mk1C platform (Oxford Nanopore Technologies, Oxford, UK) or the PacBio RS II platform with P6-C4 chemistry (Pacific Biosciences, Menlo Park, CA, USA).

For Oxford Nanopore sequencing, genomic DNA was processed using the SQK-LSK109 ligation sequencing kit (Oxford Nanopore Technologies, Oxford, UK), and barcoding was performed using the EXP-NBD104/114 Native Barcoding Expansion kits (Oxford Nanopore Technologies, Oxford, UK), allowing for multiplex sequencing on a single R9.4.1 flow cell. Barcoded libraries were pooled in equimolar concentrations prior to loading, with the goal of achieving an average genome coverage greater than 200× per sample.

Sequencing was conducted on the MinION Mk1C, and basecalling was performed using Guppy v4.2.3 in super accuracy mode (Phred score ≥ 7). Raw read quality was assessed using FastQC v0.11.5. De novo genome assembly was performed using Flye v2.9, with the parameters --nano-raw, --plasmids, --meta, --threads 8, and --genome-size 2.8 m to support assembly from raw Nanopore reads, enable plasmid detection, and accommodate uneven coverage.

For PacBio sequencing, SMRTbell libraries were prepared using the SMRTbell Template Prep Kit, and sequencing reads were processed using SMRT Analysis v2.3.0. Genome assembly was conducted using HGAP v3 within the SMRT Analysis suite.

Assembly quality for both platforms was evaluated using QUAST v5.2.0, with key parameters including N50, genome size, and GC content.

Gene prediction and annotation were performed using Prokka v1.14.6, employing the *Staphylococcus*-specific database. Functional annotations were assigned based on COG, GO, and KEGG categories. All nucleotide and protein sequences were further examined using BLAST+ v2.14.0 (NCBI, Bethesda, MD, USA). QIAGEN^®^ CLC Genomics Workbench v22.0.2 (QIAGEN, Hilden, Germany) was subsequently used for the visualization of contigs and reference-based alignment checks to support comparative inspection.

Resistant genes in the whole-genome data were identified using ResFinder with 100% identity matching to more than 1411 known resistant genes [34]. The Comprehensive Antibiotic Resistance Database (CARD, https://card.mcmaster.ca/, accessed on 16 September 2024) was used for the detection of AMR genes using the tBLASTn method [35]. Pathogenicity factors were searched using VFanalyzer against the Virulence Factor Database (VFDB) [36]. VirulenceFinder was used to identify known virulence genes in *Escherichia coli*, Enterococcus, and *S. aureus* through BLAST analysis, which supports both complete and partial genomes across different sequencing platforms [37]. Bacteriophage sequences within bacterial genomes or plasmids were identified, annotated, and visualized using the PHAge Search Tool (PHAST), which provides detailed characterization of prophage sequences [38]. SCC*mec* gene cassettes were analyzed by identifying the positions of the *rlmH*, *mec*, and *ccr* gene complexes within the full-length sequences. Homologous gene fragments were then identified using BLASTN 2.16.0 (NCBI, Bethesda, MD, USA).

The complete genome sequences of all 20 strains were deposited in the NCBI GenBank database and are publicly accessible.

## Figures and Tables

**Table 1:** Summary of molecular characteristics of 20 *S. lugdunensis* strains after WGS.

Strain ID	MLST	SCC*mec*	Oxacillin Susceptibility	Crispr Type	Lugdunin Operon	IS256	WGS Method	Plasmid
53	1	-	S	IIIA	+	−	Nanopore	-
249		-	S	IIIA	+	−	Nanopore	-
47	3	V	R	-	+	−	Nanopore	1
71		V	S	-	+	−	Nanopore	-
99		V	R	-	+	−	Nanopore	1
131		V	R	-	+	−	PacBio	2
135		Vt	S	-	+	−	Nanopore	1
138		IV	R	-	+	−	Nanopore	-
220		-	S	-	+	+	Nanopore	-
30	4	-	S	IIC	−	−	Nanopore	-
167		-	S	IIC	−	−	Nanopore	-
195		-	S	IIC	−	+	Nanopore	-
36	6	II	R	IIIA	+	+	Nanopore	-
118		II	R	IIIA	+	+	PacBio	1
29	27	V	S	IIC	−	−	Nanopore	2
35		V	S	IIC	−	−	Nanopore	-
149		Vt	R	IIC	−	−	Nanopore	-
37		-	S	IIC	−	−	Nanopore	-
210		-	S	IIC	−	−	Nanopore	-
248	29	-	S	IIC	−	−	Nanopore	-

**Table 2:** Phage sequence distribution and characteristics in *S. lugdunensis* strains by sequence type.

Strain ID	ST	Phage Sequence	Region Length	Completeness	Score	Most Common Phage	GC %
SL249	1	2	34.2 Kb	questionable	81	Staphy_PT1028_NC_007045	32.1%
			44.5 Kb	incomplete	30	Staphy_StB12_NC_020490	34.5%
SL53	1	1	34.3 Kb	questionable	81	Staphy_PT1028_NC_007045	32.3%
SL47	3	not found					
SL71	3	1	47.9 Kb	intact	94	Staphy_StB20_like_NC_028821	32.5%
SL99	3	not found					
SL131	3	not found					
SL135	3	not found					
SL138	3	not found					
SL220	3	1	20.2 Kb	incomplete	10	Staphy_PT1028_NC_007045	31.4%
SL30	4	1	27.7 Kb	incomplete	40	Staphy_PT1028_NC_007045	30.1%
		2	48.2 Kb	intact	120	Staphy_187_NC_007047	34.5%
SL167	4	1	34.3 Kb	intact	150	Staphy_187_NC_007047	29.6%
		2	27.6 Kb	incomplete	40	Staphy_PT1028_NC_007045	30.0%
		3	62.2 Kb	incomplete	50	Staphy_CNPx_NC_031241	35.1%
SL195	4	1	52.9 Kb	intact	150	Staphy_CNPx_NC_031241	35.2%
SL36	6	1	32.8 Kb	intact	110	Staphy_phiETA2_NC_008798	34.4%
		2	112.2 Kb	intact	93	Staphy_SPbeta_like_NC_029119	30.9%
SL118	6	1	110.4 Kb	intact	94	Staphy_SPbeta_like_NC_029119	30.9%
		2	33.4 Kb	incomplete	30	Staphy_StB12_NC_020490	34.4%
SL29	27	1	37.5 Kb	incomplete	60	Staphy_SPbeta_like_NC_029119	30.6%
SL35	27	1	37.5 Kb	incomplete	60	Staphy_SPbeta_like_NC_029119	30.6%
SL37	27	not found					
SL149	27	not found					
SL210	27	not found					
SL248	29	1	45.4 Kb	intact	150	Staphy_CNPx_NC_031241	35.4%

**Table 3:** Antimicrobial resistance profiles and corresponding ResFinder-predicted resistance genes across various *S. lugdunensis* strains.

Strain ID	SCC*mec*	P ^1^	FOX ^2^	CC	E	SXT	TEC	VA ^3^	ResFinder Gene
53	-	S	S	S	S	S	S	S	Not Found
249	-	S	S	S	S	S	S	S	Not Found
47	V	R	R	S	S	S	S	S	*blaZ*, *mecA*
71	V	R	S	S	S	S	S	S	*blaZ*, *mecA*
99	V	R	R	S	S	S	S	S	*blaZ*, *mecA*
131	V	R	R	S	S	S	S	S	*blaZ*, *mecA*
135	Vt	R	S	S	S	S	S	S	*blaZ*, *mecA*
138	IV	R	R	R	R	S	S	S	*blaZ*, *mecA*
220	-	R	S	R	R	R	S	S	*blaZ*, *mecA*
30	-	R	S	S	S	S	S	S	*blaZ*
167	-	R	S	S	S	S	S	S	Not Found
195	-	S	S	S	S	S	S	S	*blaZ*; *aac*(*6′*)*-aph*(*2″*)
36	II	R	R	R	R	S	S	S	*aac*(*6′*)-*aph*(*2″*); *aph*(*3′*)-*III*; *ant*(*9*)-*Ia*; *ant*(*6*)-*Ia*; *mecA*; *blaZ*; *erm*(*A*)
118	II	R	R	R	R	S	S	S	*aac*(*6′*)-*aph*(*2″*); *aph*(*3′*)-*III*; *ant*(*9*)-*Ia*; *ant*(*6*)-*Ia*; *mecA*; *blaZ*; *fusB*; *erm*(*A*)
29	V	S	S	S	S	S	S	S	*mecA*
35	V	S	S	S	S	S	S	S	*mecA*
149	Vt	R	R	S	S	S	S	S	*mecA*; *lnu*(*A*)
37	-	R	S	S	S	S	S	S	*blaZ*
210	-	S	S	S	R	S	S	S	Not Found
248	-	S	S	R	R	S	S	S	*ant*(*9*)-*Ia*; *erm*(*A*)

^1^ Antimicrobial susceptibility was determined in accordance with CLSI M100 guidelines unless otherwise noted. Penicillin (P), erythromycin (E), clindamycin (CC), trimethoprim–sulfamethoxazole (SXT), tetracycline (TEC), and cefoxitin (FOX) were tested using the disk diffusion method. ^2^ Cefoxitin was used as a surrogate to determine oxacillin (OX) susceptibility. ^3^ Vancomycin (VA) susceptibility was assessed using the agar dilution method. An MIC of <6 μg/mL was considered susceptible based on internal laboratory criteria, which differs from the CLSI M100 breakpoint (S ≤ 4 μg/mL) for non-*aureus Staphylococcus* spp.

## Data Availability

The original contributions presented in this study are included in the article. Further inquiries can be directed to the corresponding author.

## Supplementary Materials

The following supporting information can be downloaded at https://www.mdpi.com/article/10.3390/ijms26136106/s1.
