# Supplementary material for: Study of lug Operon, SCCmec Elements, Antimicrobial Resistance, MGEs, and STs of Staphylococcus lugdunensis Clinical Isolates Through Whole-Genome Sequencing
Source: Int J Mol Sci. 2025 Jun 25;26(13):6106. doi: 10.3390/ijms26136106 (PMC12250136; doi:10.3390/ijms26136106)
Supplement: Supplementary file 1 [file ijms-26-06106-s001.zip › ijms-3657340-supplementary.pdf]

Supplement Table S1. Anatomical sources and GenBank accession numbers of 20 *Staphylococcus lugdunensis* isolates obtained from the strain repository at Chang Gung Memorial Hospital.

**Strains ID**	**MLST**	**Oxacillin MIC (µg/mL)**	**Specimen type**	**Genebank/ BioSample Accession number**
53	1	0.5	Blood	SAMN48713852
249		0.5	Pus	SAMN48713862
47	3	32	Blood	SAMN48713851
71		0.5	Blood	SAMN48713853
99		>32	Blood	SAMN48713854
131		32	Blood	NZ_CP048007.1
135		1	Blood	SAMN48713855
138		16	Blood	NZ_CP048713.1
220		0.5	WD	SAMN48713860
30	4	0.5	Aspirate	SAMN48713848
167		1	Throat Swab	SAMN48713857
195		0.5	Pus	SAMN48713858
36	6	>32	Blood	NZ_CP048714.1
118		>32	Blood	NZ_CP048008.1
29	27	1	Blood	SAMN48713847
35		1	Blood	SAMN48713849
149		4	Blood	SAMN48713856
37		1	Blood	SAMN48713850
210		0.5	Throat swab	SAMN48713859
248	29	0.5	Blood	SAMN48713861
